# LLM-Enhanced Chinese Morph Resolution in E-Commerce Live Streaming Scenarios

**DOI:** 10.3390/e27070698

**Published:** 2025-06-29

**Authors:** Xiaoye Ouyang, Liu Yuan, Xiaocheng Hu, Jiahao Zhu, Jipeng Qiang

**Affiliations:** 1 China Academy of Electronic and Information Technology, Beijing 100041, China; ouyangxiaoye@cetc.com.cn (X.O.); huxiaocheng@cetc.com.cn (X.H.); 2 School of Information Engineering, Yangzhou University, Yangzhou 225127, China; mz120231031@stu.yzu.edu.cn (J.Z.); jpqiang@yzu.edu.cn (J.Q.)

**Keywords:** morph resolution, e-commerce live streaming, Large Language Models

## Abstract

E-commerce live streaming in China has become a major retail channel, yet hosts often employ subtle phonetic or semantic “morphs” to evade moderation and make unsubstantiated claims, posing risks to consumers. To address this, we study the Live Auditory Morph Resolution (LiveAMR) task, which restores morphed speech transcriptions to their true forms. Building on prior text-based morph resolution, we propose an LLM-enhanced training framework that mines three types of explanation knowledge—predefined morph-type labels, LLM-generated reference corrections, and natural-language rationales constrained for clarity and comprehensiveness—from a frozen large language model. These annotations are concatenated with the original morphed sentence and used to fine-tune a lightweight T5 model under a standard cross-entropy objective. In experiments on two test sets (in-domain and out-of-domain), our method achieves substantial gains over baselines, improving $F_{0.5}$ by up to 7 pp in-domain (to 0.943) and 5 pp out-of-domain (to 0.799) compared to a strong T5 baseline. These results demonstrate that structured LLM-derived signals can be mined without fine-tuning the LLM itself and injected into small models to yield efficient, accurate morph resolution.

## 1. Introduction

E-commerce live streaming has swiftly become one of China’s most vibrant retail channels: platforms like Douyin host over nine million live broadcasts each month and facilitate the sale of more than ten billion items through these sessions [1]. At the same time, hosts frequently employ “morphs”—subtle phonetic or semantic alterations and filler insertions—to evade real-time moderation and make unsubstantiated claims (e.g., implying medicinal properties of ordinary products). Resolving these morphs in real time is therefore essential to protect consumers and uphold industry standards [2,3].

Existing work on morph resolution has largely focused on written obfuscations in social media commentary or underground domains such as illegal gambling and adult content [4,5,6]. Those studies define morphs as text-level transformations designed to bypass keyword filters (e.g., splitting or substituting characters) and target entirely different subject areas (news, politics, vice industries). By contrast, the live-streaming context presents two key distinctions:

- **Modality and Purpose**: Morphs here occur in speech, exploiting ASR weaknesses—presenters may split characters (‘‘胡’’ (*hú*)->‘‘古月’’(*gǔ yùe*)) or insert meaningless fillers (‘‘手术’’(*shǒu shù, surgery*)->‘‘手某术’’(*shǒu mǒu shù, surgery*)) to disrupt censoring systems.
- **Domain**: Live commerce skews heavily toward health- and medical-related claims, where undetected false advertising can directly harm consumers.

In this paper, we study the Live Auditory Morph Resolution (LiveAMR) task. One recent work transformed the task into a text-to-text generation problem [7]. In the era of Large Language Models (LLMs), some works have explored the possibility of LLMs to enhance the performance of small large models [8,9,10,11]. Building upon this foundation, we propose a simple but effective LLM-enhanced training framework that mines three forms of explanation information from an off-the-shelf large language model and injects them into a lightweight LiveAMR model:

- **Morph Types.** We prompt the LLM with a predefined schema of structural and semantic morph categories. These explicit type labels help the small model narrow its correction search space.
- **Reference Corrections.** Although LLM-generated corrections are not used verbatim, they serve as intermediate “hints” that guide the small model toward more accurate outputs.
- **Natural-Language Explanations.** Constrained by rationality and comprehensiveness criteria, the LLM produces clear analyses of each morph error—offering soft supervision on why and how to correct.

We then concatenate these LLM-derived annotations with the original (noisy) input and feed the combined text into the small LiveAMR model during training and inference. This approach “mines” the LLM’s stored morph-resolution knowledge without any LLM fine-tuning, while preserving the efficiency and task alignment of supervised learning for the downstream model.

Our contributions are twofold:

- **Novel Paradigm.** We introduce the first LLM-enhanced training framework for LiveAMR, systematically extracting morph types, references, and explanations from an off-the-shelf LLM and integrating them into a small model.
- **Performance and Efficiency**. We evaluate our framework on the publicly released LiveAMR dataset [7], which consists of 6,236 positive and 76,554 negative training examples, along with both in-domain and out-of-domain test sets. We show that incorporating LLM-derived annotations—without additional LLM fine-tuning—significantly boosts small-model accuracy while keeping training costs low.

## 2. Related Work

**Morph Resolution in English:** Computational morphology in English focuses on tasks such as segmentation, lemmatization, tagging, and disambiguation. Morphological tagging assigns morphosyntactic features (e.g., *runs* → V;PRES;3P;SG) [12]. Disambiguation systems then select the correct parse from multiple analyses based on context [12,13,14]. Early approaches used rule-based or finite-state methods, while modern techniques rely on neural or statistical models. Unsupervised methods like Morfessor infer morpheme boundaries from raw text [12] and neural taggers jointly learn features and parts of speech. High-quality morphological analysis is shown to benefit downstream tasks like parsing and machine translation. These works emphasize the integration of morph analysis with other NLP tasks.

**Morph Resolution in Chinese:** Chinese differs significantly due to its lack of inflectional morphology [15]. Characters represent minimal semantic units, and words are formed by segmenting character sequences. As a result, Chinese NLP systems often employ character-based models and joint segmentation-POS tagging strategies [15]. Morph resolution in Chinese can also involve decoding slang, puns, or disguised terms—especially in online or censored contexts.

You et al. [5] use context-aware autoencoders to resolve invented morphs in Chinese social media. Zhang et al. [16] propose a context-aware entity morph decoding framework that links morphs to canonical entities. These methods rely heavily on contextual embeddings and background knowledge to resolve ambiguous or creative variants in text.

**Live Auditory Morph Resolution (LiveAMR):** Zhu et al. [7] introduce the Live Auditory Morph Resolution (LiveAMR) task to normalize speech-transcribed morphs in Chinese e-commerce live streams. They treat LiveAMR as a sequence generation task and demonstrate that LLM-generated synthetic data improves small model performance.

Our work builds on this by proposing the first LLM-enhanced training framework for LiveAMR. Instead of relying on runtime LLM inference or fine-tuning, we query an off-the-shelf LLM to extract morph types, references, and explanations, which are injected into a small model’s training process. This approach significantly improves both accuracy and efficiency—by distilling LLM knowledge without incurring its inference cost.

## 3. Method

**Figure 1:**
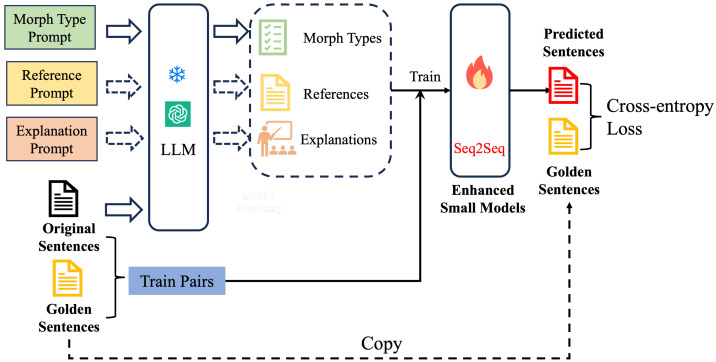
Our designed framework of the LLM-enhanced LiveAMR training pipeline.

Figure 1 illustrates our LLM-enhanced training framework for LiveAMR. First, each morphed sentence is issued three tailored prompts (Morph Type, Reference, and Explanation) to a frozen LLM in a zero- or few-shot setting, where “frozen LLM” implies its parameters are not updated during the training process of the small model. The LLM returns a morph-type label, a pseudo-label reference correction, and a natural-language rationale. These three outputs are concatenated with the original sentence to form enriched input–output pairs. Finally, we train a compact T5 model on these enhanced examples, leveraging its unified text-to-text framework for a consistent input/output representation, its powerful span-corruption pre-training to jump-start language understanding, its availability in lightweight configurations (e.g., 60M parameters) to ensure computational efficiency, and its broad community support for easy reproducibility—thereby internalizing the LLM’s knowledge for fast, accurate inference.

**Problem Definition:** Let $D={\left\{\left(x_{i},y_{i}\right)\right\}}_{i=1}^{N}$ be our annotated corpus of live-streamed Chinese sentences, where each $x_{i}$ may contain one or more *morphs*—lexical alterations introduced by hosts to evade moderation—and $y_{i}$ is the intended, de-morphed version. The LiveAMR task seeks to learn a function

f:x↦y

that recovers all original terms faithfully. Accurately modeling *f* is challenging because morphs can take diverse forms (structural, or semantic substitutions) and context matters in disambiguating them.

**Rationale for LLM-Generated Explanations:** Large language models (LLMs) trained on massive multilingual corpora encode deep knowledge of linguistic structure, phonetics, and semantics. When prompted appropriately, they can not only correct errors but also articulate the reasoning behind each correction. We exploit this capability by defining an *annotation function*

$$E\left(x\right)\;=\;\left(t,\;r,\;e\right),$$

which extracts from *x*:

- A high-level morph type *t*, narrowing the model’s focus to a specific class of error.
- A pseudo-label reference sentence *r*, providing an example “ideal” correction.
- A natural-language explanation *e*, conveying the LLM’s diagnostic reasoning.

Injecting E(x) into small-model training has two main benefits:

- Search Space Reduction: Knowing *t* limits the candidate corrections the model must consider.
- Auxiliary Supervision: The pair (r,e) serves as pseudo-labels and rationales, guiding the small model toward human-like correction strategies without expensive LLM inference during deployment.

**Explanation Information Mined from LLMs:** For each sentence *x*, we query the LLM in a zero- or few-shot prompt to compute the following:

$$t={\mathrm{LLM}}_{\mathrm{type}}\left(x\right),\;r={\mathrm{LLM}}_{\mathrm{ref}}\left(x\right),\;e={\mathrm{LLM}}_{\exp}\left(x\right).$$

where the prompt templates are shown in Appendix A.

We further elaborate each component:

Morph Types: We pre-define two non-overlapping morph categories—*structural* and *semantic*. Structural morphs include homophonic substitutions or character insertions (e.g., “白褂褂” equals “医生”), while semantic morphs replace words with near-synonyms. The LLM is constrained to choose exactly one label from {structural, semantic, none}. This explicit label *t* informs the small model of the type of transformation to apply, reducing ambiguity.

Reference Corrections: Although LLM outputs can vary, they often produce fluent and accurate corrections. We collect the LLM’s best-guess corrected sentence *r* as a pseudo-label. While *r* is not used as the ground-truth target, presenting *r* alongside the original *x* provides the small model with a concrete exemplar of how the morphs should be resolved, acting as soft guidance.

Explanations: We prompt the LLM to articulate a rationale *e* for each correction, subject to three quality criteria:

- Rationality: The explanation must be coherent and written in clear, natural Chinese.
- Comprehensiveness: All morphs present in *x* should be addressed.
- Specificity: Each erroneous term in *x* must be directly linked to its corrected form.

By including *e*, we transfer the LLM’s diagnostic insight into the training data, enabling the small model to learn not only *what* the correction is, but also *why*.

**Training Process of Small Modeling:** We build an enriched input sequence

$$\tilde{x}\;=\;\left[\mathrm{Type}:\right]\;t\;∥\;\left[\mathrm{Ref}:\right]\;r\;∥\;\left[\mathrm{Exp}:\right]\;e\;∥\;\left[\mathrm{Orig}:\right]\;x,$$

where “||” denotes string concatenation with delimiters. This concatenation ensures the model receives all LLM-provided signals in a structured format.

We fine-tune a pre-trained T5 encoder–decoder model $f_{\theta }$ on $\left\{\left({\tilde{x}}_{i},y_{i}\right)\right\}$ by minimizing the cross-entropy loss

$$L\left(\theta \right)=-\sum_{i=1}^{N}\log P_{\theta }\left(y_{i}|{\tilde{x}}_{i}\right).$$

**Inference Process:** At test time, for an unseen sentence *x*:

- Compute (t,r,e)=E(x) via the LLM prompts.
- Construct $\tilde{x}=\left[\mathrm{Type}:\right]\;t\;∥\;\left[\mathrm{Ref}:\right]\;r\;∥\;\left[\mathrm{Exp}:\right]\;e\;∥\;\left[\mathrm{Orig}:\right]\;x$.
- Generate the final correction using the fine-tuned T5 model.
  $$\hat{y}=\arg\max_{y}P_{\theta }\left(y|\tilde{x}\right),$$

This pipeline ensures that at inference, no LLM fine-tuning or heavy computation is required beyond the initial annotation step, enabling efficient, accurate LiveAMR resolution in production settings.

## 4. Experiment

### 4.1. Experimental Setup

**Metrics.** The model is expected to change only the morphological elements in the target sentences, leaving all other components untouched. Evaluation is conducted at the sentence level with strict criteria: a positive sample counts as correct only if all morphological forms are accurately restored, while a negative sample is considered correct only if the model leaves the sentence completely unchanged. We choose three common metrics to evaluation: Precision, Recall, and $F_{0.5}$.

**Dataset.** The dataset [7] is chosen for training and evaluation. The statistical information of the dataset is shown in Table 1. It includes two Tests (Test1 and Test2), where Test set 1 and the training set are from the same live room, and test set 2 and the training set are from different live rooms.

**Baselines.** The following models were selected as the baseline for comparison:

(1) Large Language Models (LLMs): To assess the effectiveness of LLMs in resolving morphological ambiguities, three prominent models known for their Chinese language understanding capabilities were chosen: GPT-3.5-turbo (https://openai.com/ (accessed on 2 January 2025)), Deepseek-V2 (https://platform.deepseek.com/ (accessed on 2 January 2025)), and GLM4-Plus (https://chatglm.cn/ (accessed on 2 January 2025)). These models are all large, pre-trained transformer-based architectures. Eight examples from the training dataset—comprising six positive and two negative instances—were manually chosen and incorporated into the prompts as contextual examples. The temperature setting was consistently fixed at 0.7.

(2) Seq2Seq Models: Convseq2seq [17] and BART [18] were chosen as the backbone architectures for sequence-to-sequence modeling and fine-tuned.

(3) Kenlm and Seq2Edit: To further demonstrate that sequence-to-sequence models are more appropriate for the morph resolution task, the statistical language model Kenlm [19] and the BERT-based editing model Seq2Edit [20] were also chosen.

(4) **T5 and DataAug**: T5 and DataAug [7] were based on T5 (mengzi-T5-base [21]). In addition to using the annotated training data, DataAug also leverages data constructed by an LLM to augment the training set and participates in model training. The newly added morph dataset contains 11,280 positive samples and 2155 negative samples.

(5) Our method: The small modeling is the same as the T5 and DataAug methods. The LLM modeling is called by OpenAI’s API, and the version of LLM is “gpt-4o-mini-2024-07-18”, chosen for its balance of performance and efficiency in generating high-quality explanations. During the training process, the maximum length of the input sequence is set to 128, and the initial learning rate is set to $1\times 10^{-4}$. We train the model for 20 epochs on a 24GB Nvidia 3090Ti GPU with the batch size set to 32. We use the AdamW optimizer, and the model employs a cosine annealing learning rate schedule.

### 4.2. Results Analysis

Table 2 compares our LLM-enhanced framework against a variety of baselines on both Test1 (in-domain) and Test2 (out-of-domain). Several key observations emerge as follows:

These models (GPT, Deepseek, GLM) achieve moderate precision but suffer from low recall on Test1 (e.g., GPT: p=0.421, R=0.320, $F_{0.5}=0.364$; Deepseek: p=0.660, R=0.529, $F_{0.5}=0.587$). On Test2, recall improves slightly (up to ≈0.649 for GLM), but precision remains inconsistent, yielding sub-60% $F_{0.5}$ scores. This indicates that generic LLMs—though knowledgeable—are not specialized enough for robust morph resolution without task-specific fine-tuning.

KenLM attains reasonable precision (p≈0.61) but very low recall (R≈0.37), reflecting its inability to generate novel corrections. Seq2Edit achieves near-perfect precision (p>0.96) by only performing high-confidence edits, but its recall remains below 0.37, leading to $F_{0.5}\approx 0.53$. Both methods underscore that surface-level edits or n-gram scoring alone are insufficient for comprehensive morph recovery.

ConvSeq2Seq yields extremely high precision (p>0.97) but moderate recall (R≈0.53), giving $F_{0.5}=0.685$ on Test1 and dropping to 0.573 on Test2. BART balances precision and recall more evenly (p=0.701, R=0.767, $F_{0.5}=0.738$ on Test1; $F_{0.5}=0.639$ on Test2). These results confirm that pre-trained generative models better capture diverse morph patterns but still exhibit domain sensitivity.

A vanilla T5 model fine-tuned on the annotated dataset achieves high precision (0.950) and decent recall (0.656) on Test1 ($F_{0.5}=0.872$) but suffers a severe recall drop to 0.334 on Test2. DataAug, which incorporates LLM-generated pseudo-examples into training, raises recall to 0.774 on Test1 ($F_{0.5}=0.907$) and partially mitigates the drop on Test2 (R=0.417, $F_{0.5}=0.745$). This demonstrates that data-level augmentation alone can improve generalization but has limited impact on out-of-domain recall.

On Test1, our method attains the highest precision (0.975) and recall (0.799), yielding $F_{0.5}=0.943$, which represents a +7.1 gain over T5 and +3.6 over DataAug. Crucially, on the out-of-domain Test2, we achieve R=0.501 and $F_{0.5}=0.799$, outperforming T5 by +26.0 and DataAug by +5.4 in $F_{0.5}$. These gains highlight that structured LLM-derived signals—morph types, reference corrections, and detailed explanations—provide robust, generalizable guidance that improves morph resolution under domain shift.

In summary, our LLM-enhanced training framework consistently delivers superior precision-first $F_{0.5}$ performance across both in-domain and out-of-domain tests, validating the effectiveness of mining and injecting LLM explanations into small-model training.

### 4.3. Ablation Study

Table 3 reports an ablation of our LLM-enhanced framework on Test1, measured by $F_{0.5}$. Augmenting the base T5 model with LLM-predicted morph-type labels yields a substantial jump in $F_{0.5}$ from 87.20 to 93.70 (+6.50). This highlights that explicit morph-type information drastically narrows the model’s search space, enabling more accurate and targeted corrections.

Incorporating natural-language explanations on top of morph types provides a modest further gain (from 93.70 to 93.90, +0.20). The small increase indicates that rationales help the model refine its decisions, but much of the benefit has already been captured by knowing the error class.

Finally, including the LLM-generated reference corrections boosts $F_{0.5}$ to 94.34 (+0.44 over explanations). Reference pseudo-labels offer concrete exemplars of the desired output, giving the model additional guidance beyond abstract explanations.

Overall, the ablation study shows that each annotation type contributes positively: morph-type labels yield the largest improvement, while explanations and references offer incremental yet complementary benefits. Together, they enable our small T5 model to achieve state-of-the-art precision-first performance on LiveAMR.

## 5. Conclusions

We propose an LLM-enhanced training framework for Live Auditory Morph Resolution, mining morph-type labels, reference corrections, and natural-language rationales from a frozen LLM and injecting them into a compact T5 model. By concatenating these annotations with the original input and training under a standard supervised objective, our method internalizes LLM reasoning without fine-tuning. Experiments on in-domain and out-of-domain tests show substantial $F_{0.5}$ gains (0.943 and 0.799), outperforming strong baselines. While our method demonstrates significant improvements, certain limitations warrant consideration for future research. The performance is inherently dependent on the quality and consistency of the LLM-generated annotations; any inaccuracies or biases in the LLM’s output could propagate. Future work will explore multilingual extensions and additional LLM-derived supervision, aiming to mitigate these limitations and further enhance the generalizability and robustness of LiveAMR systems.

## Figures and Tables

**Table 1:** The statistics of the constructed Chinese morph dataset.

	Positive and Negative	Morph Num
Train	6236/76,554	7301
Valid	800/800	1025
Test1	800/800	1081
Test2	400/400	548

**Table 2:** The results of different methods. The best results are bolded; “*” indicates p<0.05 (bootstrap resampling against the T5 baseline).

	Test1			Test2		
Method	Pre	Recall	$F_{0.5}$	Pre	Recall	$F_{0.5}$
GPT	0.421	0.320	0.364	0.494	0.441	0.466
Deepseek	0.660	0.529	0.587	0.667	0.626	0.646
GLM	0.484	0.515	0.499	0.525	0.649	0.580
Kenlm	0.607	0.372	0.537	0.515	0.513	0.514
Seq2Edit	0.968	0.361	0.526	0.987	0.408	0.588
Convseq2seq	0.978	0.527	0.685	0.898	0.421	0.573
BART	0.701	0.767	0.738	0.670	0.611	0.639
T5	0.950	0.656	0.872	**0.936**	0.334	0.539
DataAug	0.948	0.774 *	0.907 *	0.927 *	0.417	0.745 *
Our method	0.975 *	0.799 *	0.943 *	0.935 *	0.501 *	0.799 *

**Table 3:** Ablation results on Test1.

Method	$F_{0.5}$
T5-base	87.20
+ Morph Type	93.70
+ Morph Type + Explanation	93.90
+ Morph Type + Explanation + Reference	94.34

## Data Availability

No new data were created or analyzed in this study. Data sharing is not applicable to this article.

## Appendix A

### Prompt Templates

Below are the three core prompt templates and the final LLM-enhaned prompt template used to query the frozen LLM. In each case, the {fewshot} placeholder is filled by retrieved examples (via BM25) and the {source_sentence}, {target_sentence}, and {reference_sentence} placeholders by the current data record.

**Table A1:** Prompt for Morph Types (with English annotations for clarity).

Role	Content
System	变体词还原是将句子中的所有变体词还原为其原词, 非变体词不进行任何修改。
	*(English: Morph restoration requires converting all morphs in the sentence back to their original words; non-morph tokens should remain unchanged.)*
	变体词类型主要包含两种：{改善：该某善，问题：小问小题, 提高：提什么高}（结构变体词）
	和{眼睛：心灵之窗, 酒：8+1, 医生：白褂褂, 糖尿病患者：小糖人, 一百元：一百米}（语义变体词）。
	*(English: There are two morph categories: structural morphs (e.g., “*该某善*” for “*改善*”; “*小问小题*” for “*问题*”; “*提什么高*” for “*提高”*) and semantic morphs (e.g., “*心灵之窗*” for “*眼睛*”; “8+1” for “*酒*”; “*白褂褂*” for “*医生*”; “*小糖人*” for “*糖尿病患者*”; “*一百米*” for “*一百元”*).)*
	注意可能存在ASR错误。
	*(English: Note that some tokens may result from ASR transcription errors.)*
	这是一些相关的例子: {fewshot}
	*(English: Here are some related examples: {fewshot}.)*
User	根据用户输入的原句和参考句，生成所需内容。
	*(English: Given the user’s original sentence and the reference sentence, generate the required output.)*
	用户原句: <source_sentence>
	*(English: Original sentence: <source_sentence>)*
	还原后内容: <restored_content>
	*(English: Restored content: <restored_content>)*
	参考句: <reference_sentence>
	*(English: Reference sentence: <reference_sentence>)*
	输出目标使用<target></target>包裹；解释使用<explanation></explanation>包裹。
	*(English: Wrap the output in <target></target>; wrap the explanation in <explanation></explanation>.)*

**Table A2:** Prompt for Reference Corrections (with English annotations).

Role	Content
System	变体词还原是将句子中的所有变体词还原为其原词, 非变体词不进行任何修改。
	*(English: Morph restoration requires converting all morphs in the sentence back to their original words; non-morph tokens should remain unchanged.)*
	变体词类型主要包含两种：{改善：该某善，问题：小问小题, 提高：提什么高}（结构变体词）
	和{眼睛：心灵之窗, 酒：8+1, 医生：白褂褂, 糖尿病患者：小糖人, 元/钱：米}（语义变体词）。
	*(English: There are two morph categories: structural morphs (e.g., “*该某善*” for “*改善*”; “*小问小题*” for “*问题*”; “*提什么高*” for “*提高*”) and semantic morphs (e.g., “*心灵之窗*” for “*眼睛*”; “8+1” for “*酒*”; “*白褂褂*” for “*医生*”; “*小糖人*” for “*糖尿病患者*”; “*米*” for “*元”*).)*
	注意可能存在ASR转录错误。
	*(English: Note that some tokens may result from automatic speech recognition (ASR) errors.)*
	这是一些相关的例子: {fewshot}
	*(English: Here are some related examples: {fewshot}.)*
User	你需要对用户输入的句子进行变体词还原。
	*(English: You need to restore any morphs in the user’s input sentence.)*
	用户输入: <source_sentence>
	*(English: User input: <source_sentence>)*
	如果不存在变体词则不修改，直接输出。
	*(English: If there are no morphs, leave the sentence unchanged.)*
	输出结果使用<target></target>包裹。
	*(English: Wrap the output sentence with <target></target>.)*

**Table A3:** Prompt for Explanations (with English annotations).

Role	Content
System	变体词还原是将句子中的所有变体词还原为其原词，非变体词不进行任何修改。
	*(English: Restore all morphs in the sentence to their original words; leave non-morph tokens unchanged.)*
	变体词类型主要包含两种：结构变体词{改善：该某善，问题：小问小题，提高：提什么高}，
	以及语义变体词{眼睛：心灵之窗，酒：8+1，医生：白褂褂，糖尿病患者：小糖人，元/钱：米}。
	*(English: There are two morph categories: structural morphs (e.g., “*该某善*” for “*改善*”; “*小问小题*” for “*问题*”; “*提什么高*” for “*提高”*) and semantic morphs (e.g., “*心灵之窗*” for “*眼睛*”; “8+1” for “*酒*”; “*白褂褂*” for “*医生*”; “*小糖人*” for “*糖尿病患者*”; “*米*” for “*元”*).)*
	注意：部分变体词可能是由ASR转录错误或口语表达引起的，这些不是变体词。
	*(English: Note: Some alterations may result from ASR errors or colloquial speech and are not true morphs.)*
	这是一些相关的例子: {fewshot}
	*(English: Here are some related examples: {fewshot}.)*
User	你需要对用户输入的句子结合所给的参考句，生成用户指定的解释。
	*(English: Given the user’s input sentence and the provided reference, generate the specified explanation.)*
	用户输入source: <source_sentence>
	*(English: User input (source): <source_sentence>)*
	这是目标句子：<target_sentence>
	*(English: Target sentence: <target_sentence>)*
	输出的解释使用<explanation></explanation>包裹。
	*(English: Wrap the explanation in <explanation></explanation> tags.)*
	解释格式为："变体词"的原词是"原词"，如："米"的原词是"元"。
	*(English: Explanation format: “<morph>”’s original word is “<original>”, e.g., “米”’s original word is “元”.)*
	不要输出错误类型！
	*(English: Do not output the error type!)*

**Table A4:** Prompt for LLM-enhanced explanations.

Role	Content
System	变体词还原...（同上）
	*(English: Variant word normalization... (same as above) )*
	这是一些相关的例子: {fewshot}
	*(English: Here are some relevant examples: {fewshot} )*
User	根据用户输入的原句和参考句，生成类型、解释和参考句。
	*(English: Based on the user’s input sentence and reference sentence, generate the type, explanation, and reference sentence. )*
	用户输入 source: <source_sentence>
	*(English: Based on the user’s input sentence and reference sentence, generate the type, explanation, and reference sentence. )*
	类型使用<type></type>包裹，解释使用<explanation></explanation>包裹，
	*(English: Wrap type with <type></type>, explanation with <explanation></explanation>, )*
	参考句使用<target></target>包裹。
	*(English: Wrap reference sentence with <target></target>. )*
	如果无变体词，则type=“不存在变体词”，解释=“无”，参考=原句。
	*(English: If there is no variant word, then type = "No variant word", explanation = "None", reference = original sentence. )*
